# Dynamics of Erythropoietic Biomarkers in Response to Treatment With Erythropoietin in Belgrade Rats

**DOI:** 10.3389/fphar.2018.00316

**Published:** 2018-04-10

**Authors:** Ly M. Nguyen, Aman P. Singh, Pawel Wiczling, Wojciech Krzyzanski

**Affiliations:** ^1^Department of Pharmaceutical Sciences, The State University of New York at Buffalo, Buffalo, NY, United States; ^2^Department of Biopharmaceutics and Pharmacodynamics, Medical University of Gdańsk, Gdańsk, Poland

**Keywords:** anemia, iron utilization, red blood cell survival, DMT1, pharmacokinetics, pharmacodynamics

## Abstract

Recombinant human erythropoietin (rHuEPO) is used effectively in the treatment of various anemic disorders. Belgrade rat is a useful animal model of anemia caused by defect in iron utilization. The objective of the present study was to investigate the dynamics of erythropoietic biomarkers in Belgrade rats receiving rHuEPO. Pharmacokinetics of rHuEPO was evaluated in Belgrade rats and normal rats after intravenous administration of single doses of the drug (100 and 1350 IU/kg). Pharmacodynamic biomarkers included levels of red blood cells, hemoglobin, and reticulocytes following administration of a single intravenous dose of rHuEPO (100 IU/kg). Red blood cell survival was assessed after treatment with rHuEPO (450 IU/kg), three times a week for 2 weeks. It was found that rHuEPO exhibited non-linear pharmacokinetics in both Belgrade and control rats. At the low dose, plasma concentrations and AUC (area under the curve) were significantly lower while clearance and volume of distribution were higher in Belgrade rats (*p* < 0.05). At the higher dose, there was no difference in pharmacokinetics between the two groups. Erythropoietic effect of rHuEPO was negligible in Belgrade rats at the dose of 100 IU/kg whereas all studied erythropoietic biomarkers were increased in normal rats. The levels of red blood cells, hemoglobin were significantly lower whereas the percentage of reticulocytes was higher in Belgrade rats compared to that in normal rats (*p* < 0.05). RHuEPO increased red blood cell survival in both animal groups. In conclusion, rHuEPO effect on erythropoietic biomarkers was stronger in normal rats than Belgrade rats at the studied doses. The findings from this study may provide further insights into understanding of anemic disorders resulting from mutations in the divalent metal transporter.

## Introduction

Belgrade (b/b) rats originate from X-irradiated albino rats in Belgrade, Yugoslavia (Sladic-Simic et al., 1966). These rats have a hereditary hypochromic microcytic anemia. The newborn anemic rats are pale yellow with growth retardation and easily distinguished from the normal littermates. Peripheral blood smears exhibit significant microcytosis, anisocytosis, and poikilocytosis which are exacerbated with age. The levels of RBC and HGB are significantly decreased and progressively decline over time (Sladic-Simic et al., 1966). In addition, a decrease in platelets and leukocytosis are also observed in these animals (Ivanovic, 1997).

The cause of anemia in Belgrade rats is attributed to a mutation in the DMT1. A glycine-to-arginine substitution in the fourth transmembrane domain of DMT1 causes loss of activity of the transporter (Fleming et al., 1998). DMT1 plays a major role in intestinal iron absorption and in the transferrin iron delivery cycle. In systemic circulation, iron is carried by transferrin and iron bound transferrin is endocytosed into endosomes through the transferrin cycle. Defective DMT1 impairs transport of iron into the cytosol of erythroid progenitor cells leading to decrease in HGB synthesis, and thus anemia (Garrick L.M. et al., 1999; Garrick M.D. et al., 1999).

Recombinant human erythropoietin (30.4 kDa) stimulates erythropoiesis by binding to and activating erythropoietin receptors expressed on erythroid progenitor cells in the bone marrow, leading to proliferation, differentiation, and maturation of these cells into mature RBCs (Elliott et al., 2008). RHuEPO effectively improves anemia by increasing RET count, RBC count, HGB level, and HCT. Its effect in rats is well-documented (Woo et al., 2007; Ait-Oudhia et al., 2010). In humans, it has revolutionized anemia treatment in patients with chronic renal disease, AIDS, or cancer patients undergoing chemotherapy (Elliott et al., 2008). Since iron is an essential component of HGB, effectiveness of rHuEPO treatment is enhanced by combining with iron supplementation in patients subjected to iron deficiency (Van Wyck, 1989).

Belgrade rats with their inherent anemia and iron disorder resemble characteristics of anemia observed in humans. Patients with hypochromic microcytic anemia caused by mutations in the gene encoding DMT1 have been identified in the last decade (Iolascon et al., 2008; Bardou-Jacquet et al., 2011; Casale et al., 2018). These patients appear to respond to rHuEPO treatment even though iron utilization by erythroid precursors is not improved. In most of the cases iron overload is present indicating functional iron deficiency (Iolascon et al., 2008). In CKD patients with anemia, iron treatment is used to compensate for iron losses due to hemodialysis, chronic bleeding, and frequent phlebotomy (Babitt and Lin, 2012). RHuEPO treatment often fails in 10–20% of CKD patients and one of the main reasons is functional iron deficiency caused by sequestration of iron in the liver and reticuloendothelial cells (Ganz and Nemeth, 2012; Hung et al., 2014). Therefore, studying the effects of rHuEPO in Belgrade rats has significant implications in clinical practice.

In addition to increasing RBC production, rHuEPO has been shown to prolong RBC survival. In chronic hemodialysis patients, RBC survival increases significantly under rHuEPO treatment, but after cessation of the therapy it decreases to the pretreatment values (Polenakovic and Sikole, 1996). In patients with chronic renal failure and uremia, similar results are also observed (Schwartz et al., 1990). However, the mechanism of prolonging RBC survival is not clear. To date, we are not aware of any studies on the effect of rHuEPO on RBC survival in animals or humans with defect in iron utilization due to mutation in DMT1.

The purpose of this report is to investigate the dynamics of erythropoietic biomarkers in Belgrade rats after treatment with rHuEPO. We aim to evaluate possible differences in RBC, HGB, RET, and RBC survival between Belgrade rats and normal heterozygous (+/b) rats. We also present results of studies addressing rHuEPO pharmacokinetics in Belgrade rats in comparison with the control animals.

## Materials and Methods

### Animals

Control heterozygous rats and Belgrade rats were bred and maintained in our laboratory. The rats were used in this study when they were at least 12 weeks old. The control rats were fed a standard diet without iron supplementation, whereas Belgrade rats were fed an iron supplemented diet (ferrous sulfate heptahydrate supplemented rat chow, Krackeler Scientific, Inc., Albany, NY, United States) necessary for their survival. The animals were kept in a quiet room on a 12/12-h light/dark cycle with free access to food and water. The study protocol was approved by the Institutional Animal Care and Use Committee of the State University of New York (Buffalo, NY, United States).

### Materials

Recombinant human erythropoietin (EPOGEN^®^ 2000 IU/mL, Amgen, Thousand Oaks, CA, United States) is a 165 amino acid glycoprotein produced by mammalian cells transfected with the human erythropoietin gene using the recombinant DNA technology. Other materials which were used in this study include isotonic saline (0.9% sodium chloride injection, Baxter, Deerfield, IL, United States), bovine serum albumin lyophilized powder (Sigma-Aldrich, St. Louis, MO, United States), EDTA (2%) (Sigma-Aldrich, St. Louis, MO, United States), isoflurane (Piramal Healthcare Limited, India), DPBS (Thermo Fisher Scientific, Waltham, MA, United States), RPMI Medium 1640 (Thermo Fisher Scientific, Waltham, MA, United States), biotin (Sigma-Aldrich, St. Louis, MO, United States), dimethyl sulfoxide (Themo Fisher Scientific, Waltham, MA, United States), R-phycoerythrin (Thermo Fisher Scientific, Waltham, MA, United States), and Retic-Count Reagent (BD Biosciences, San Jose, CA, United States).

### Selection of rHuEPO Doses

The rHuEPO doses used in all of the studies were selected based on the calculation that the clearance of rHuEPO is approximately three times faster in rats than in humans (Krzyzanski et al., 2005; Woo et al., 2006, 2007). As a result, the 100 and 1350 IU/kg doses are approximately equivalent to 30 and 450 IU/kg doses in humans, respectively. These doses represent a low dose (in CKD patients) and a high dose (in cancer patients) used in clinical practice. Similarly, the 450 IU/kg dose is equivalent to a 150 IU/kg dose in humans which is a high dose used in CKD patients.

### Pharmacokinetic Study and Analysis

Both control rats and Belgrade rats (*n* = 5) were injected with a single IV dose of rHuEPO via tail vein. The doses of rHuEPO (100 and 1350 IU/kg) were prepared in isotonic saline solutions with 0.25% bovine serum albumin before injection. Blood samples were collected via tail vein at 0, 0.08, 0.5, 1, 4, 12, 24, and 48 h after injection. The rats were anesthetized using 5% isoflurane before injection or blood collection. EDTA (2%) was used to prevent blood coagulation. The blood samples were centrifuged within 30 min of collection at 1000 *g* for 15 min to extract plasma samples which were then stored at -20°C in a non-self-defrosting freezer for quantitative assay of rHuEPO.

Non-compartmental analysis of the plasma concentration – time data was performed using Phoenix 64 WinNonlin^®^ 7.0 (Certara L.P., Princeton, NJ, United States). Calculation of pharmacokinetic parameters included AUC, C_max_, C_max_D_, CL, V_ss_, and t_1/2_.

### Pharmacodynamic Study

To avoid excessive blood collection in each animal the pharmacodynamic study was conducted on a separate occasion and in different groups of animals which were not used in the pharmacokinetic study. Both control rats and Belgrade rats (*n* = 5) were injected with a single IV dose of rHuEPO 100 IU/kg via tail vein. The doses of rHuEPO were prepared in isotonic saline solutions with 0.25% bovine serum albumin before injection. Blood samples were collected via tail vein at 0, 1, 2, 3, 4, 7, 9, and 11 days after injection. The rats were anesthetized using 5% isoflurane before injection or blood collection. EDTA (2%) was used to prevent blood coagulation. Pharmacodynamic effects of rHuEPO were evaluated by measuring hematological parameters including HGB, RBC, HCT, MCH, MCV, monocyte count, and RET within 4 h of the blood sample collection.

### Red Blood Cell Survival Study

The effect of rHuEPO on RBC survival was studied in control and Belgrade rats at the dose of 450 IU/kg, three times a week for 2 weeks (t.i.w. × 2). This regimen was expected to stimulate production of a significant amount of RBCs enough to observe the effect of rHuEPO on RBC survival. To determine baseline RBC survival, the placebo group (2 +/b rats and 2 b/b rats) were given isotonic saline. To evaluate the effect of rHuEPO, the treatment group (2 +/b rats and 2 b/b rats) were given rHuEPO 450 IU/kg via tail veins, t.i.w. × 2. The RBC survival was determined using the method based on biotinylation of RBCs (Hoffmann-Fezer et al., 1993). Briefly, 2.5 mL of blood was collected via tail vein for each rat using EDTA (2%) to avoid coagulation. The whole blood was centrifuged and RBCs were separated and washed four times with DPBS. After that, cells were suspended in RPMI Medium 1640. The suspension was then incubated at 37°C with addition of biotin dissolved in dimethyl sulfoxide. Biotinylated RBCs were washed three times with DPBS and suspended to make a 50% HCT suspension using RPMI Medium 1640. A volume of 2.5 mL of this suspension was reinfused via tail vein into the same rat from which the original blood was obtained. Blood samples were taken after 1 h, and 24 h thereafter daily for the first week, and three times a week until the measured signal reached the limit of quantification. Biotinylated RBCs were detected by streptavidin conjugated to R-phycoerythrin analyzed by flow cytometry (FACSCalibur, BD Biosciences, San Jose, CA, United States).

### RHuEPO Plasma Concentration Assay

The plasma concentrations of rHuEPO were determined using the Quantikine IVD erythropoietin enzyme-linked immunosorbent assay (ELISA) kit (R&D Systems, Minneapolis, MN, United States) following the manufacturer’s instructions. The assay was specific for rHuEPO, thus, preventing cross reaction with endogenous rat EPO. The standard curve was constructed using standard concentrations ranging from 0 to 200 mIU/mL. Any plasma samples, potentially containing rHuEPO above 200 mIU/mL, were diluted with the diluent provided by the manufacturer. The lower limit of detection of the assay was <0.6 mIU/mL and the coefficient of variation was <10%. All the samples were analyzed on the same day to prevent inflated variability.

### Hematological Assay

Hematological measurements including HGB (g/dl), RBC (10^9^ cells/mL), MCH (pg/cell), HCT (%), MCV (fL), monocyte count (10^9^ cells/L), and RBC distribution width (RDW, %) were determined using BC-2800Vet Auto Hematology Analyzer (Mindray Bio-Medical Electronics, Co., Ltd., Shenzhen, China). RET (%) was measured by flow cytometry (FACSCalibur, BD Biosciences, San Jose, CA, United States) following staining with Retic-Count Reagent. All procedures were performed following the manufacturer’s instructions.

### Statistical Analysis

Tukey’s *post hoc* tests were used after performing the two-way repeated measures ANOVA to evaluate the statistical significance for the comparisons of the means of rHuEPO plasma concentration, HGB, RBC, MCH, and RET between the control rats and Belgrade rats at each time point. Similarly, comparisons between the baseline means and the means at each time point within each group were performed using the one-way repeated measures ANOVA method and Tukey’s *post hoc* tests. Comparisons of pharmacokinetic parameters between the two groups of rats or between two doses of rHuEPO were carried out using Wilcoxon rank sum tests. All the analyses were carried out by the MIXED or NPAR1WAY procedure (SAS 9.4, SAS Institute, Inc., Cary, NC, United States).

## Results

### Pharmacokinetics

**FIGURE 1:**
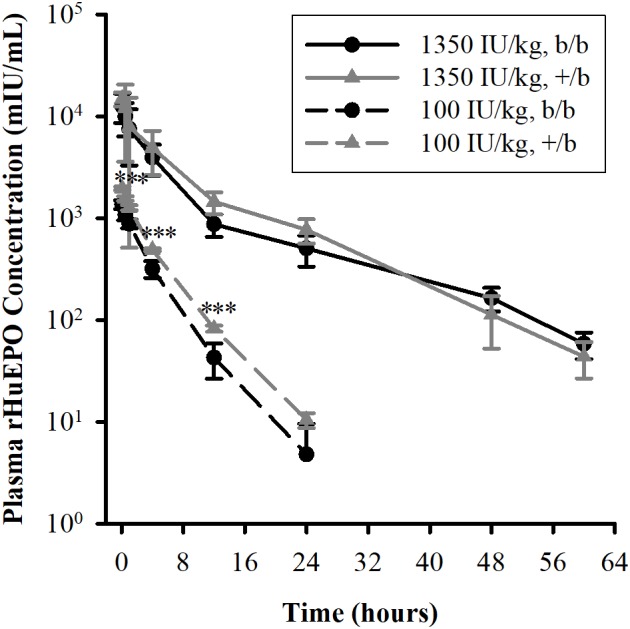
Plasma concentrations of rHuEPO after an IV dose in Belgrade (b/b, black solid circle) and control (+/b, gray solid triangle) rats. Data are presented as mean ± standard deviation (*n* = 4–5). Between group comparison of plasma concentration at each time point was performed using Tukey’s *post hoc* test in conjunction with two-way repeated measures ANOVA. Significant differences in comparisons between b/b and +/b groups given the same dose are indicated as follows: ^∗^*p* < 0.05, ^∗∗^*p* < 0.01, and ^∗∗∗^*p* < 0.001. For the 100 IU/kg dose, significant differences were observed at all time points except at 24 h. For the 1350 IU/kg dose, no differences were observed at all time points.

The mean plasma concentrations versus time profiles in b/b and +/b rats after receiving an IV dose of rHuEPO are presented in **Figure 1**, and pharmacokinetic parameters are summarized in **Table 1**. At the low dose of 100 IU/kg, there were significant differences between the two groups regarding AUC, C_max_, CL, and V_ss_, but no difference in t_1/2_. At the higher dose of 1350 IU/kg, only difference in t_1/2_ was observed. Within group comparisons showed that, in the b/b group, C_max___D_ and CL were not significant different, whereas V_ss_ and t_1/2_ were significantly higher for the 1350 IU/kg dose (*p* < 0.05). In the +/b group, C_max___D_, V_ss_, and t_1/2_ were different between the two dosing groups (*p* < 0.05), but CL was not. Overall, the pharmacokinetic data suggested non-linear pharmacokinetics of rHuEPO in the dose range 100–1350 IU/kg in both b/b and +/b rats. Additionally, there were differences in pharmacokinetic parameters between the two groups of rats.

**Table 1:** Pharmacokinetic parameters in b/b and +/b rats at two different IV doses of rHuEPO.

Parameter	100 IU/kg (*n* = 5)		1350 IU/kg (*n* = 4)	
	b/b	+/b	b/b	+/b
AUC (mIU.h/mL)	4085 ± 713^∗∗^	6367 ± 192	64159 ± 21637	78284 ± 24879
C_max_ (mIU/mL)	1431 ± 148^∗∗^	2056 ± 92	13754 ± 4520	16814 ± 1985
C_max___D_ (kg/mL)	0.014 ± 0.0015^∗∗^	0.021 ± 0.00092^#^	0.010 ± 0.0033	0.012 ± 0.0015
CL (mL/h/kg)	25 ± 3.9^∗∗^	16 ± 0.47	23 ± 9.6	19 ± 5.7
V_ss_ (mL/kg)	90 ± 7.1^∗∗^^#^	67 ± 3.9^#^	269 ± 139	213 ± 135
t_1/2_ (h)	3.0 ± 0.58^#^	3.6 ± 0.17^#^	12.2 ± 0.82^∗^	9.0 ± 1.3

*Data are presented as mean ± standard deviation. AUC, total area under the plasma concentration-time curve; C_max_, maximum concentration; C_max_D_, dose-normalized C_max_; CL, clearance; V_ss_, volume of distribution; t_1/2_, terminal half-life. Significant differences in comparisons using Wilcoxon rank sum tests are indicated as follows: between b/b and +/b groups given the same dose: ^∗^p < 0.05, ^∗∗^p < 0.01, and ^∗∗∗^p < 0.001; between two doses within each animal group: ^#^p < 0.05, ^##^p < 0.01, ^###^p < 0.001.*

### Pharmacodynamics

**FIGURE 2:**
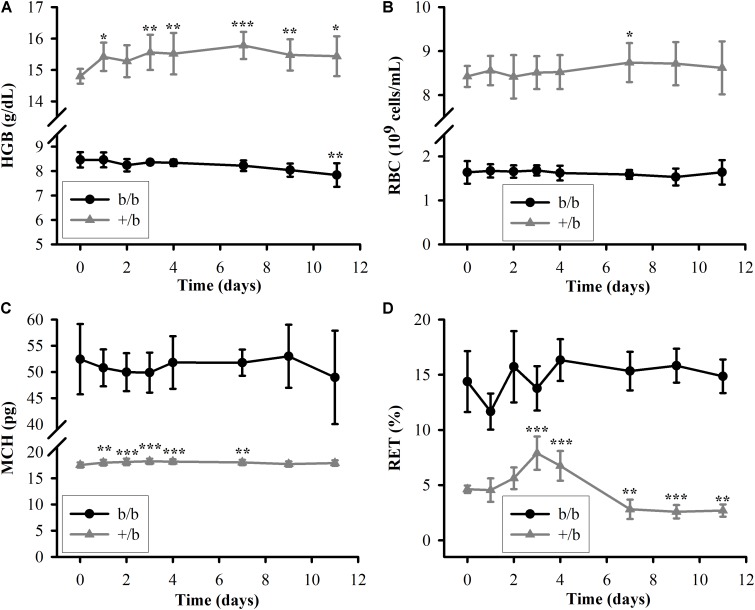
Pharmacodynamic responses in Belgrade rats (b/b, black solid circle) and control rats (+/b, gray solid triangle) after a single IV dose of rHuEPO 100 IU/kg (*n* = 3–5). **(A–D)** The data are presented as mean ± standard deviation for HGB, RBC, MCH, and RET. Between groups comparisons of the biomarkers at each time point were performed using Tukey’s *post hoc* tests in conjunction with two-way repeated measures ANOVA. Within each group, comparisons of the biomarkers between later time points and the baseline (day 0) were performed using Tukey’s *post hoc* tests in conjunction with one-way repeated measures ANOVA. The levels of HGB, RBC, MCV, and RET in the b/b group are significantly different from that in the +/b group at all time points (*p* < 0.001). Significant differences from the baseline within each animal group are indicated as follows: ^∗^*p* < 0.05, ^∗∗^*p* < 0.01, and ^∗∗∗^*p* < 0.001.

The measurements of HGB, RBC, MCH, and RET in b/b and +/b rats before and after a single IV dose of rHuEPO 100 IU/kg are presented in **Figure 2**. It was clear that the differences between the Belgrade rats and control rats in all four markers at each time points were significant (*p* < 0.001). For HGB (see **Figure 2A**), the mean baseline concentration in the control group almost doubled that in the Belgrade group (14.8 ± 0.23 vs. 8.5 ± 0.31 g/dL). During the course of the study, the HGB concentration in the control group increased to a plateau (around 15.5 ± 0.66 g/dL on day 3) and maintain this level, whereas in the Belgrade group it decreased steadily from day 1 (8.5 ± 0.30 g/dL) to day 11 (7.8 ± 0.48 g/dL). As for RBC counts (see **Figure 2B**), the measurements were about fivefold higher in the control rats than in Belgrade rats (e.g., 8.5 ± 0.37 vs. 1.7 ± 0.12 10^9^ cells/mL on day 3) at all time points (*p* < 0.001). The RBC count increased in the control rats but it was not significant compared to the baseline, except for day 7 (*p* < 0.05). In the Belgrade rats, the RBC count did not decrease steadily as seen in the HGB concentration and was not significantly different from the baseline (*p* > 0.05). In contrast to the results observed for HGB and RBC where the measurements in the control group were greater than in the Belgrade group, the MCH level (see **Figure 2C**) in the Belgrade rats was about threefold higher than that in the control rats (e.g., 51.8 ± 4.0 vs. 18.2 ± 0.46 on day 4) at all time points (*p* < 0.001). The MCH level in Belgrade rats after rHuEPO dosing was not different compared to the baseline (*p* > 0.05), while in the control rats it increased to a plateau (around 18.2 ± 0.52 on day 3) and then decreased after several days. For the last pharmacodynamic response, the percentage of RET (see **Figure 2D**), the measurements in the Belgrade group were greater than that in the control group at all time points (*p* < 0.001). In the first 4 days, the percentage of RET was highly variable in the Belgrade rats before becoming more stable thereafter. In contrast, in the control rats RET increased and peaked around day 3 (7.9 ± 1.5%). After that it decreased below the baseline (*p* < 0.01) on day 7 (2.8 ± 0.9%) and maintained this level to the last day of the study.

### Red Blood Cell Survival

**FIGURE 3:**
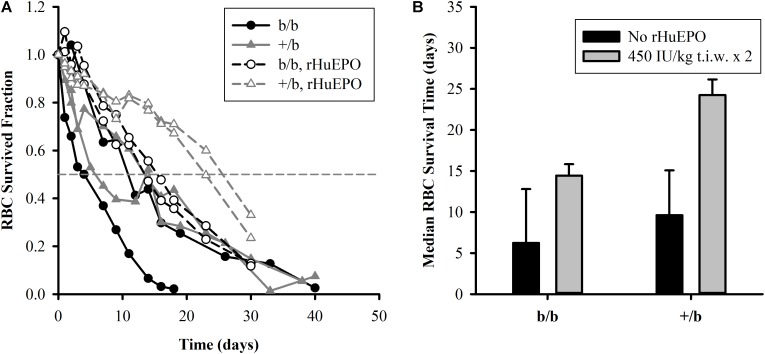
(**A**, left panel) Individual plots of RBC survival in Belgrade rats (b/b) and control rats (+/b) for the placebo group (solid symbols) and the treatment group (open symbols) with rHuEPO 450 IU/kg, three times a week for 2 weeks. The horizontal dashed line represents RBC survived fraction of 0.5. (**B**, right panel) Median survival time of RBCs in the Belgrade rats (b/b) and control rats (+/b) for the placebo group (black bars) and in the treatment group (gray bars). No statistical tests were performed due to small sample size.

The RBC survival before and after the IV administration of rHuEPO 450 IU/kg (t.i.w. × 2) is presented in **Figure 3**. The results show that, in the placebo group (*n* = 2 for each subgroup), b/b rats had higher variability in RBC survival compared to +/b rats with one b/b rat expressing much lower RBC survived fraction than the other at all time points. One of the b/b rats had similar RBC survival to that of the +/b rats. Since the sample size was small, the results did not show a clear difference between b/b and +/b rats in the placebo group. For the treatment group (*n* = 2 for each subgroup), at the early time, there was no difference in RBC survival between the two rat groups. The RBC survival times at the survived fraction of 0.9 were about the same, from 3 to 5 days. After that, the +/b rats had higher survived fraction (days 10 to 30) (see **Figure 3A**). The median survival time were 14.4 ± 1.4 and 24.3 ± 1.9 days for b/b and +/b rats, respectively (see **Figure 3B**). Within b/b rats, for most of the time points, the RBC survived fraction was higher in the rats receiving rHuEPO. The rHuEPO treatment also produced an increase of 131% (from 6.2 to 14.4 days) in the median RBC survival time. This effect was smaller compared to that in +/b rats where the corresponding increase was 152% (from 9.6 to 24.3 days) (see **Figure 3B**). Additionally, for all time points the RBC survived fraction for the +/b rats was higher in the treated group than in the untreated group. Overall, rHuEPO had slightly stronger effect on RBC survival in +/b rats than in b/b rats.

## Discussion

### Pharmacokinetics

Previous studies show that rHuEPO exhibits non-linear pharmacokinetics in rat (Kinoshita et al., 1992; Kato et al., 1997), monkey (Woo et al., 2007), and human (Veng-Pedersen et al., 1995; Woo et al., 2007). These studies demonstrate that the non-linear pharmacokinetics is attributed to the saturation of the EPO receptor-mediated elimination pathway in the bone marrow leading to a decrease in clearance when rHuEPO doses increase. In our study, even though clearance is not different between the two dosing groups, non-linear pharmacokinetics is implied by the increase in volume of distribution and terminal half-life in both b/b and +/b rats. Studies in Belgrade rats show that erythropoietic activity is diminished, manifested by a low number of erythroid progenitors and decreased cellular proliferation (Pavlovic-Kentera et al., 1989; Biljanovic-Paunovic et al., 1992). These progenitor cells require EPO for their survival and are the targets for rHuEPO (Sawada et al., 1987; Broudy et al., 1991). Consequently, Belgrade rats exhibit significantly lower levels of HCT, hence, higher plasma volumes. Results from non-compartmental analysis show that, at the 100 IU/kg dose, b/b rats have lower plasma concentrations and drug exposure (AUC), but higher volume of distribution and clearance compared to normal rats. It should be noted that volumes of distribution obtained from non-compartmental analysis may be indicative of non-linear pharmacokinetics, but they do not reflect the true extent of distribution of rHuEPO because non-compartmental analysis assumes linear pharmacokinetics while rHuEPO follows non-linear pharmacokinetics. Lower plasma concentrations and AUC can be explained by a significant increase in plasma volume by approximately 87% in b/b rats due to lower levels of HCT (6.6 ± 1.1 vs. 49.4 ± 0.6, *p* < 0.001, at baseline levels, **Table 2**). It was suggested that rHuEPO can be degraded and eliminated by the cells of the mononuclear phagocyte system (Elliott et al., 2008). Consistent with this effect the lower levels of plasma concentrations and AUC could be partially explained by the significant increase in the level of monocytes in b/b rats compared to +/b rats (1.0 ± 0.41 vs. 0.18 ± 0.08 10^9^ cells/L, *p* < 0.05, at baseline levels, **Table 2**).

**Table 2:** Baseline levels of monocyte, absolute reticulocyte (RET), hematocrit (HCT), mean corpuscular volume (MCV), and red blood cell distribution width (RDW).

Group	Monocyte (10^9^ cells/L)	Absolute RET (10^9^ cells/mL)	HCT (%)	MCV (fL)	RDW (%)
b/b rats	1.0 ± 0.41^∗∗^	0.16 ± 0.048^∗^	6.6 ± 1.12^∗∗^	40.7 ± 0.52^∗∗^	21.0 ± 1.06^∗∗^
+/b rats	0.18 ± 0.08	0.23 ± 0.018	49.4 ± 0.60	58.7 ± 1.73	10.1 ± 0.60

*Data are presented as mean ± standard deviation, n = 4. Significant differences in comparisons between b/b and +/b groups using Wilcoxon rank sum tests are indicated as follows: ^∗^p < 0.05, ^∗∗^p < 0.01, and ^∗∗∗^p < 0.001.*

At the 1350 IU/kg dose, even though the differences were not significant, lower plasma concentrations and AUC, higher volume of distribution and clearance were also observed in Belgrade rats. This is expected because at a significantly higher dose, elimination of rHuEPO through the receptor-mediated pathway is saturated. As a result, the non-saturable elimination pathway, such as proteolysis by various enzymes in blood and tissues, would be mainly responsible for rHuEPO clearance and this pathway may not be different between b/b and +/b rats. However, it should be noted that the variability of the pharmacokinetic parameters at the 1350 IU/kg dose is considerably higher compared with that at the 100 IU/kg dose (see **Table 1**). Challenges in breeding Belgrade rats resulted in limited supply of animals and made our studies underpowered. The differences between the two groups of animals may be observed when sample size is increased.

### Pharmacodynamics

Recombinant human erythropoietin has been shown to improve anemia in experimental animals and humans. It stimulates erythropoiesis by binding to and activating EPO receptors on the membrane of the erythroid precursors in the bone marrow. This interaction stimulates cell proliferation and differentiation into RETs and then RBCs. In the absence of EPO, precursor cells undergo apoptosis (Koury and Bondurant, 1988; Kelley et al., 1994). In our study, the 100 IU/kg rHuEPO treatment does not have significant hematopoietic effect on b/b rats. The levels of RET, RBC, HGB, and MCH do not increase over time after dosing. In contrast, the corresponding measurements increase significantly in +/b rats (see **Figure 2**). The rHuEPO effect on +/b rats is expected as it was demonstrated in previous studies in healthy rats (Woo et al., 2007). Selection of higher doses for our study could have resulted in a resolution of the hematopoietic response markers. Belgrade rats exhibit microcytic and hypochromic anemia (Sladic-Simic et al., 1966) due to impaired iron uptake and iron utilization caused by a mutation of the DMT1. This transporter is mainly responsible for transport of iron across cell membranes (Fleming et al., 1998). Erythropoiesis in Belgrade rats is compromised with a low number of erythroid progenitors, decreased cellular proliferation, and abnormal cell morphology. As a result, Belgrade rats have low levels of RET, RBC, HGB, and other abnormal hematological parameters (Pavlovic-Kentera et al., 1989; Biljanovic-Paunovic et al., 1992). These characteristics may help to explain the unresponsiveness of Belgrade rats to rHuEPO.

The results from the RBC survival study indicate that rHuEPO increases RBC survival in both animal groups. Our data suggest that the increased fraction of younger RBCs after rHuEPO treatment is responsible for the increase in RBC survived fraction. Previous studies in humans propose that rHuEPO effect on erythroid precursors stabilizes cell membrane and ultimately increases RBC cell viability (Schwartz et al., 1990; Polenakovic and Sikole, 1996). According to the initial endowment concept stating that RBC senescence is mainly a function of the cell status during its progenitor stage (Landaw, 1988), rHuEPO might also affect other cell characteristics that make it fitter for survival. Additionally, we observe that rHuEPO is slightly more effective in increasing RBC survived fraction in +/b rats than in Belgrade rats at the given dose of 450 IU/kg, t.i.w. × 2. Our study was underpowered to test if this difference was significant.

In agreement with previous studies, the percentage of RETs (14.3 ± 2.8%) is in the reported range (Garrick et al., 1997). It is significantly higher in b/b rats than in +/b rats as shown in **Figure 2D**. This may give an impression that b/b rats have higher levels of RET count. However, since b/b rats have much lower levels of RBC, the absolute RET counts are slightly lower compared to +/b rats (0.16 ± 0.05 vs. 0.23 ± 0.02 10^9^ cells/mL, *p* < 0.05, at baseline levels, **Table 2**). Higher percentage of RET can be attributed to the lower RBC level which is the consequence of low RET count and shorter RBC survival.

It is interesting to observe that the level of MCH in b/b rats is approximately threefold higher than +/b rats, whereas the levels of HGB, RBC, and MCV are lower (see **Figure 2** and **Table 2**). The observed levels of MCH (52.4 ± 6.7 pg, at baseline level) are significantly higher compared to previous studies (5–15 pg) (Garrick et al., 1997) while the MCV marker is similar. Although, different studies may use different methods to measure this marker, the discrepancy is substantial. The reason for this is due to the low RBC level (1.64 ± 0.25 10^9^ cells/mL) while the HGB level is comparable to previous studies. We observed the skewed distribution of RBC volume with a considerably long heavy right tail (see Supplementary Figure 2) and higher RDW in b/b rats compared with that in normal rats (RDW: 21.0 ± 1.06 vs. 10.1 ± 0.60%, *p* < 0.001, **Table 2**). This means the proportion of RBCs with volumes below MCV dominates the proportion with volumes above MCV leading to inflated MCH measurements. Although not confirmed in our study, RBC survival seems to be shorter in Belgrade rats than in normal animals. It is possible that RBCs with lower hemoglobin content are preferentially removed from the circulation by the reticuloendothelial system resulting in skewed distribution of cells with higher corpuscular hemoglobin. This would explain both lower RBC counts and higher MCH observed in Belgrade rats in our study. RBC survival dependence on animal age and disease progression would account for discrepancies with previously reported results.

## Conclusion

We have characterized pharmacokinetics of rHuEPO and the dynamics of erythropoietic biomarkers including RBC, HGB, RET, and RBC survival in Belgrade rats. Pharmacokinetics of rHuEPO was shown to be non-linear. At the high dose (1350 IU/kg), there was no difference between the Belgrade rats and control rats. At the low dose (100 IU/kg), plasma concentrations and AUC were lower in Belgrade rats. The treatment of rHuEPO at the low dose (100 IU/kg) showed no significant erythropoietic effect on Belgrade rats. In contrast, rHuEPO significantly increased erythropoietic biomarkers in the control rats. Belgrade rats had lower levels of RBC and HGB, higher percentage of RET. RHuEPO increased RBC survival in both animal groups. The findings from this study may provide further insights into understanding of anemic disorders resulting from mutations in DMT1.

## Author Contributions

WK: conceived the study, designed the experiments, and wrote the paper. LN: performed the experiments, analyzed data, and wrote the paper. AS and PW: performed the experiments and edited the paper.

## Conflict of Interest Statement

The authors declare that the research was conducted in the absence of any commercial or financial relationships that could be construed as a potential conflict of interest.

## Abbreviations

AUC: total area under the plasma concentration-time curve
CKD: chronic kidney disease
CL: clearance
C_max_: maximum concentration
C_max_D_: dose-normalized C_max_
DMT1: divalent metal transporter
DPBS: Dulbecco’s phosphate buffer saline
EDTA: ethylenediaminetetraacetic acid
EPO: erythropoietin
HCT: hematocrit
HGB: hemoglobin
MCH: mean corpuscular hemoglobin
IV: intravenous
MCV: mean corpuscular volume
RBC: red blood cell
RET: reticulocyte, RDW, red blood cell distribution width
rHuEPO: recombinant human erythropoietin
t.i.w.: three time a week
t_1/2_: terminal half-life
V_ss_: volume of distribution
