# Supplementary material for: Dynamics of Erythropoietic Biomarkers in Response to Treatment With Erythropoietin in Belgrade Rats
Source: Front Pharmacol. 2018 Apr 10;9:316. doi: 10.3389/fphar.2018.00316 (PMC5902559; doi:10.3389/fphar.2018.00316)
Supplement: Supplementary file 1 [file Data_Sheet_1.DOCX]

**Supplementary Figure 1 Legend:** Red blood cell (RBC) volume distribution at baseline in a Belgrade rat (A, left panel) and a control rat (B, right panel). Horizontal axis represents RBC volume (fL) and vertical axis represents density. The distributions at later times after a single intravenous dose of rHuEPO 100 IU/kg are similar to that at baseline.
