# Supplementary figures and images for: Dynamics of Erythropoietic Biomarkers in Response to Treatment With Erythropoietin in Belgrade Rats
Source: Front Pharmacol. 2018 Apr 10;9:316. doi: 10.3389/fphar.2018.00316 (PMC5902559; doi:10.3389/fphar.2018.00316)

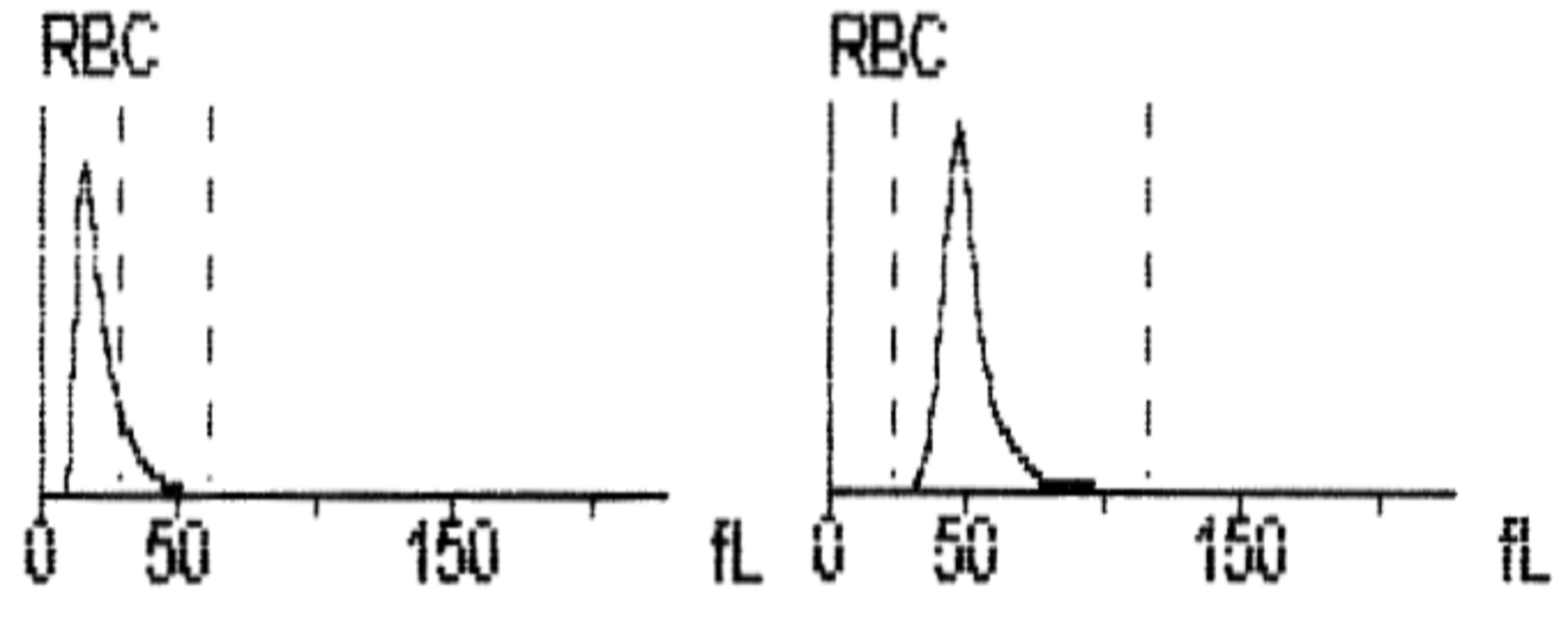

Supplement: Supplementary file 2 [file Image_1.TIF]
